# Flourishing, Affect, and Relative Autonomy in Adult Exercisers: A Within-Person Basic Psychological Need Fulfillment Perspective

**DOI:** 10.3390/sports6020048

**Published:** 2018-05-25

**Authors:** Zişan Kazak Çetinkalp, Marc Lochbaum

**Affiliations:** 1 Faculty of Sport Science, Ege University, Bornova, Izmir 35100, Turkey; 2 Department of Kinesiology & Sport Management, Texas Tech University, Lubbock, TX 79409, USA; marc.lochbaum@ttu.edu; 3 Faculty of Sports and Health Education, Lithuania University of Educational Sciences, Vilnius 08106, Lithuania

**Keywords:** Self-Determination Theory, cluster analysis, human growth, exercise autonomy, motivation, exercise adherence

## Abstract

Flourishing is a construct used to understand human growth. Exercise psychology research is scant concerning this valuable construct. Hence, our purpose was to examine different levels of flourishing and related constructs within a large group of self-reported exercisers from a basic psychological need profile perspective. Participants were 389 female and 387 male adults attending fitness centers. Hierarchical cluster analyses revealed the presence of three clusters with significantly different psychological need profiles across the three basic needs. Separate multivariate analyses of variance were used for the analyses for our demographic variables and psychological variables. Follow-up post hoc tests showed that these clusters differed significantly and were low to moderate in meaningfulness regarding exercise min/week and sports experience. The clusters differed significantly, with moderate to large meaningfulness, in flourishing, positive affect, and relative autonomy. Self-reported exercise and sports participation were not the important cluster characteristics. Our results indicated that self-reported levels of flourishing, positive affect, and autonomy differ even within a large group of self-reported exercisers attending fitness centers that on average exceeded the weekly-recommended number of moderate-to-vigorous activity minutes. Thus, our results suggest the importance of fitness centers in meeting their participants’ three basic needs.

## 1. Introduction

Without question, decades of research tell us that not nearly enough people engage in regular exercise even with the knowledge that regular exercise is beneficial for physical and psychological functions for all persons. Thus, enhancing exercise behavior and factors that impact exercise participation is a critical issue. Though not a new construct, flourishing is used to understand human growth, especially across Europe and the Russian Federation [1]. Typically, flourishing is thought to reflect self-reported thoughts of one’s life going well [1]. Thus, flourishing is the sum of feeling good and functioning effectively [2]. However, exercise psychology research is scant concerning this valuable construct. Hence, our purpose was to bring this important construct to the exercise psychology literature by examining flourishing and related constructs more often researched. We achieved our purpose by surveying a large group of self-reported exercisers attending fitness centers from a psychological need profile perspective specific to exercising.

Self-determination Theory (SDT) is one of, if not the most, researched and promising theories for which to better understand exercise behavior [3]. The theory consists of several mini-theories that deal with the role that the contexts, interpersonal processes and individual differences, and psychological needs (basic or domain-specific) play in determining human behavior. The underlying assumption of SDT is that innate psychological needs guide human behavior. These psychological needs are autonomy, competence, and relatedness. The degree to which these three needs are satisfied will determine the type of goals a person has, the regulatory processes one chooses to use, and behavioral persistence. SDT proposes that satisfaction of three basic psychological needs is necessary for psychological growth, integrity, and well-being [3], which are the focus of this investigation within the domain of exercising. When these three needs are satisfied, individuals are more likely to be motivated to move toward situations or initiate, sustain, and maintain behavior [4].

The need for autonomy involves the individual striving to experience one’s actions as initiated by the self. Therefore, in other words, this need refers to the experience of behaving in accord with one’s interests or values [5] and feeling internal assent concerning one’s behavior [6]. Deci and Ryan [3], based on the work of White [7], define the need for competence as individuals’ inherent desire to feel effective or competent in dealing with the environment. The third need, relatedness, is the experiences of having satisfying and supportive social relationships [8]. People satisfy the relatedness need when they experience a sense of communion and develop close relationships with significant others [3]. Deci and Ryan [3] have highlighted that the fulfillment of all three needs is required to maintain or improve psychological health.

Exercise psychology researchers have demonstrated that the satisfaction of basic psychological needs is essential for positive outcomes in physical activity contexts (e.g., [9,10]). Deci and Ryan [3] suggested that satisfaction of the three basic psychological needs is required to enhance psychological well-being. Researchers [11,12] have found that satisfaction of basic needs was associated positively with psychological well-being. Likewise, researchers have demonstrated that, in exercise settings, when personal fitness-related constructs meet autonomy, competence, and relatedness needs the level of person’s motivation to exercise and psychological well-being increased [13,14]. Similarly, in the sports domain, Reinboth and colleagues [15] found that the satisfaction of the need for competence was the most important predictor of psychological and physical well-being.

Another important aspect associated with psychological need satisfaction is the affective components of subjective well-being: positive affect (PA) and negative affect (NA). PA and NA reflect the frequency and intensity of pleasant and unpleasant feelings that people experience in their lives. Low levels of NA and high levels of PA are important for subjective well-being [16]. Many researchers [11,17,18] have reported that the psychological need satisfaction was positively related to positive affect and negatively related to negative affect. Researchers [19] also found that competence and autonomy were associated positively with positive affect. In contrast, Podlog and his colleagues [19] reported that relatedness was associated negatively with negative affect. 

The basic psychological needs fuel a person’s participation reasons for a given activity [4]. Deci and Ryan [20,21] emphasized that the fulfillment of basic psychological needs in any environment, such as exercise or sport, promotes the internalization of behavioral regulation and increases well-being. Most of the studies in the physical activity contexts have supported the relationship between competence and autonomy and self-determined motivation [4,12,18,22]. Thus, environments that fulfill these domain-specific needs will facilitate both intrinsic motivation and the internalization of external motivation [23].

### Study Purpose and Hypotheses

Our main purpose was to explore whether the satisfaction of the three basic psychological needs results in different psychological health indices (flourishing, positive and negative affect, and relative autonomy) in an exercise fitness environment. Based on the tenets of SDT and past research, we hypothesized that levels of flourishing, negative and positive affect, and relative autonomy in exercise domains would differ according to the level of self-reported satisfaction of the three basic psychological needs. For instance, participants who report being very satisfied with the three basic psychological needs will have greater psychological health compared to the lesser satisfied participant, even though all participants are currently physically active. Given that basic need satisfaction predicts determined behaviors, we also tested whether fulfillment of basic need satisfaction impacted the following behavioral outcomes: minutes of exercise per week, total sports experiences, and body composition (i.e., body mass index, BMI).

## 2. Materials and Methods

### 2.1. Participants

Participants were 776 self-reported exercisers (389 female and 387 male) with a mean age of 27.99 years (range = 17–70, standard deviation (SD) = 8.45 years) recruited from a variety of exercise fitness centers in Izmir, Turkey. Participants, on average, self-reported having a healthy body mass index (BMI) of 23.30 ± 3.25 kg/m^2^ and being active, as they reported an average of 3.84 (SD = 1.31) days and mean (M) = 265.78 (SD = 152.19) minutes per week of intentional physical activity. The participants reported approximately seven years of sports experiences (M = 6.89, SD = 6.37 years) before completing the study survey.

### 2.2. Measures

#### 2.2.1. The Basic Psychological Needs in Exercise Scale (BPNES)

The BPNES, developed by Vlachopoulos and Michailidou [24] and translated into Turkish by Vlachopoulos et al. [25], assessed the basic psychological needs of the participants in exercise settings. The scale measures autonomy (e.g., “I feel that the way I exercise is definitely an expression of myself”), competence (e.g., “I feel that exercise is an activity in which I do very well”), and relatedness (e.g., “I feel very much at ease with the other exercise participants”). This scale consists of 12 items. The participants responded on a 5-point Likert-type scale using anchors of 1 (strongly disagree) and 5 (strongly agree). The higher scores indicate that needs are more fulfilled. The scale’s fit index values, calculated for the adaptation of the model, were well fit. Cronbach’s alpha values for autonomy, competence, and relatedness were 0.78, 0.72, and 0.81, respectively [25]. In the present study, the internal consistency coefficients found for the BPNES subscales were α = 0.76 for autonomy, α = 0.81 for competence, and α = 0.79 for relatedness.

#### 2.2.2. Flourishing Scale (FS)

The FS scale, developed by Diener et al. [26] and translated into Turkish by Telef [27], estimated the participants’ self-perceptions of their flourishing in life. This scale consists of eight items describing positive human functioning such as positive relationships, feelings of competence, and meaning and purpose in life (e.g., “I lead a purposeful and meaningful life”). Participants responded to each question on a 7-point scale that ranged from 1 (strongly disagree) to 7 (strongly agree). The total score may range between 8 and 56. Higher scores reflect a higher level of psychological flourishing. The reliability and validity of the profile for 529 Turkish university students were reported in a study carried out by Telef [27]. The coefficient of internal consistency in the present study was α = 0.85.

#### 2.2.3. Positive and Negative Affect Schedule (PANAS)

The PANAS [28] was translated into Turkish by Gençöz [29]. The scale, which has 20 items, was used to assess positive affect (PA) and negative affect (NA). Ten emotion words assess each affect (e.g., PA: interested, excited, strong; NA: distressed, upset, guilty). Participants rated each emotion on a 5-point Likert scale ranging from 1 (very slightly or not at all) and 5 (extremely). Scores can range from 10–50, with higher scores representing higher levels of positive and negative affect. The participants were specifically asked to rate the extent to which they have experienced each particular emotion in exercise contexts over the past two weeks (i.e., ‘‘please, while assigning a rank to each item, think about how you felt about yourself during the last two weeks’’). The construct validity and internal reliability of the PANAS have been successfully demonstrated [29]. The internal consistency coefficients found for the PANAS subscales in the present study were α = 0.80 and α = 0.81 for negative and positive affect, respectively.

#### 2.2.4. Behavioral Regulation in Exercise Questionnaire-2 (BREQ-2)

The BREQ-2 [30], translated from English to Turkish by Ersöz, Aşçı, and Altıparmak [31] was used to assess relative autonomy in exercise. The questionnaire utilizes 19 items to measure the following five subscales: intrinsic motivation, identified regulation, introjected regulation, external regulation, and amotivation. Following the stem “Why do you exercise?”, participants indicated their agreement with each item on a 5-point Likert-type scale ranging from 0 (not true for me) to 4 (very true for me). In the factor analysis carried out by Ersöz, Aşçı, and Altıparmak [31], the items were gathered differently from the original subscale. Therefore, for the present investigation, we reexamined the BREQ-2 factor structure. The fit index values, calculated for the model, supported a good fit, the root mean square error of approximation (RMSEA) = 0.04, the standardized root mean square residual (SRMR) = 0.06, the goodness of fit index (GFI) = 0.94, the non-normed fit index (NNFI) = 0.90, the comparative fit index (CFI) = 0.93). Validity and reliability evidence has been provided in support of the scale’s factor structure and internal consistency. In the present study, Cronbach’s α coefficients were 0.77 for intrinsic motivation, 0.63 for identified regulation, 0.74 for introjected regulation, 0.66 for external regulation, and 0.67 for amotivation. Then, to assess relative autonomy or self-determined motivation, the Relative Autonomy Index (RAI) was calculated using the following formula: (amotivation × (−3)) + (external regulation × (−2)) + (introjected regulation × (−1)) + (identified regulation × 2) + (intrinsic regulation × 3). The maximum score for the RAI is +20 and the minimum score is −24. Less autonomous motivation is indicated by lower negative scores on the RAI. Higher autonomous motivation is indicated by positive scores for the RAI. In this study, the index ranged from −4 to 19.

### 2.3. Procedures

We followed the first author’s human subject procedures as stipulated by her university’s committee overseeing human research. The first author obtained permission from the directors of fitness centers to recruit volunteers to participate in this study. The first author greeted potential participants at the fitness center entrances. We informed all potential participants about the current study via a written document and their rights to decide to participate voluntarily. The written materials also contained the scales to be completed. Participants who volunteered completed the questionnaire packet before they initiated their exercise program for the given day. The total time taken from participants being greeted to their completion of the questionnaire packet was approximately 15 min. Data were collected across both weekdays and weekends.

### 2.4. Data Analysis

IBM SPSS Statistics version 20 was the software used for all data analyses. Firstly, a check for outliers occurred. We defined an outlier as being standardized residual values above −3 and +3. Secondly, data are reported as means and standard deviations, and correlations among study variables were analyzed using Pearson correlations. Thirdly, we ran a hierarchical cluster analysis of the standardized z-scores for the three basic needs to identify profiles. Ward’s method and Euclidean distance evaluated groups in an analysis of variance type of approach [32]. After the hierarchical cluster analysis, K-means cluster analysis, a non-hierarchical cluster approach validated the clusters obtained from the hierarchical analysis. Finally, a multivariate analysis of variance (MANOVA) was used to evaluate the basic need fulfillment profiles with other variables. The Ryan-Einot-Gabriel-Welsh Q (REGWQ) post hoc test was the follow-up test for potential univariate F differences in the basic need fulfillment profiles. In order to interpret meaningfulness, we examined partial eta-squared (*η*^2^) and Hedges’ *g* values. We followed Cohen’s [33] interpretation guidelines for effect sizes. Hedge’s *g* of 0.20 was considered small, 0.50 medium, 0.80 large, and 1.20 or greater was considered very large and *η*^2^ interpretations of 0.01 were considered small, 0.06 moderate, and 0.14 large [34]. All analyses were conducted using a significance level of 0.05.

## 3. Results

### 3.1. Descriptive Statistics and Inter-Correlations

Table 1 contains the mean, standard deviation, and inter-correlations values for study variables. In general, the exercisers reported high scores in basic psychological needs, flourishing, PA, and RAI. In contrast, the score for NA was low. The basic psychological needs were positively related to flourishing, PA, and RAI. The NA was negatively associated with all other variables (*p* < 0.01).

### 3.2. Psychological Need Profiles

To identify the number of clusters, we considered dendrograms and the distance coefficients in the agglomeration schedule table created by the cluster analysis. According to these results, three profiles emerged from the hierarchical cluster analysis that were all significantly different (Wilks’ λ = 0.15, F (6, 1542) = 400.3, *p* < 0.001, *η*^2^ = 0.61) from each other with very meaningful differences, as indicated by Hedges’ g values for all comparisons (see Table 2). We labeled the first cluster as the “very satisfied” group. There were 233 participants in this cluster, with participants characterized by high autonomy, competence, and relatedness. The greatest number of participants (*n* = 361) fell into the second cluster labeled “satisfied”, as they were high in autonomy and competence, and moderate in relatedness. The third cluster was labeled as “moderately satisfied”, given their moderate levels of all three basic needs. This cluster consisted of 182 exerciser participants. Table 2 contains descriptive statistics for the three cluster profiles.

### 3.3. Cluster Differences in Age, BMI, Per Week Minutes of Exercise, and Total Sport Experience

We utilized MANOVA to examine whether the cluster profiles differed according to age, BMI, minutes of exercise per week, and total sports experiences. The results showed that the three clusters differed significantly, though differences were small in meaningfulness regarding minutes of exercise per week and total sports experiences (Wilks’ λ = 0.96, F (8, 1540) = 3.94, *p* < 0.001, *η*^2^ = 0.02). Test of between-subjects effects indicated that significant differences existed for minutes of exercise per week and total sports experiences. Table 2 contains these results. REGWQ post hoc tests examined the pairwise comparison between the three clusters. We calculated between-group effect size values for all possible comparisons. Results indicated that exercisers from the “very satisfied” cluster (i.e., cluster 1) had significantly (*p* < 0.05) higher scores compared to the exercise participants from the “moderately satisfied” cluster in their minutes of exercise per week. This difference was small in meaningfulness. No significant differences were observed between clusters 1 with 2 and between clusters 2 to 3. The participants in cluster 1 self-reported significantly (*p* < 0.05) higher levels of total sports experience than the participants in clusters 2 and 3. The difference between the participants in clusters 1 and 3 approached moderate meaningfulness. In addition, regarding total sports experience, the participants in cluster 2 had significantly (*p* < 0.05) higher scores than the participants in cluster 3.

### 3.4. Cluster Differences in Flourishing, PA, NA, and RAI

To explore the differences between the three clusters regarding flourishing, PA, NA, and RAI, a MANOVA was our main analysis. The results showed that the three clusters differed significantly and this significant difference was large in meaningfulness (Wilks’ λ = 0.64, F (8, 1540) = 47.86, *p* < 0.001, *η*^2^ = 0.20). Then, we examined REGWQ post hoc analyses (see Table 2). Accordingly, the exercise participants in cluster 1, the “very satisfied” cluster, self-reported significantly (*p* < 0.05) higher levels of flourishing, PA, and RAI variables than the exercise participants in the “satisfied” (i.e., cluster 2) and “moderately satisfied” (i.e., cluster 3) clusters. Also, the participants in cluster 2 reported significantly (*p* < 0.05) higher scores in these three variables compared to cluster 3. Regarding NA, the exercise participants from the “satisfied” (cluster 2) and the “moderately satisfied” (cluster 3) had significantly higher scores than those in cluster 1 (“very satisfied”). No differences existed between participants in cluster 2 and cluster 3. Except for the NA differences, all cluster differences were moderate to very large in meaningfulness, suggesting the strong importance of the three needs being vital for our main outcome variables, flourishing, PA, and RAI.

## 4. Discussion

The objective of this study was to investigate whether the need fulfillment profiles specific to exercising impacted important indicators of human functioning: flourishing, affect, relative autonomy, and physical activity behavior. Given that all participants were health club members, we hypothesized that satisfaction of exercise-related psychological needs would lead to positive, yet most likely small, physical activity behavioral outcome differences in adult exercisers. This result occurred given the significant and small to moderate differences between cluster 1 and the other two clusters for our physical activity variables. Concerning our psychological variables, the results showed that cluster 1, defined as “very satisfied” according to basic need satisfaction levels, self-reported higher scores than the other two cluster groupings in flourishing, PA, and RAI levels. On the contrary, when considering the NA, our results revealed that this group had lower scores than the other groups. These findings are in accord with Self-Determination Theory and the extant exercise psychology literature.

Regarding minutes of exercise per week and total sport experiences of participants in more detail, the exercise group defined as “satisfied” self-reported higher scores than the other exercise groups. Exercisers from cluster 1 reported more sport experiences than the other two clusters. In considering the minutes of exercise per week, the exercise group defined as “satisfied” reported longer exercise times than the exercise group labeled as “moderately satisfied”. Gourlan, Trouilloud, and Sarrazin [35] reported that three psychological needs were significantly related to physical activity length. Martinez et al. [36] reported that moderate and vigorous levels of physical activity positively correlated with perceived competence, perceived autonomy, and perceived relatedness, as they pertain to an individual’s motivation to exercise. Our results supported past literature, even with all participants being health club members.

Deci and Ryan [21] proposed that fulfilling psychological needs is associated with greater well-being. In addition, Deci and Ryan [37] also suggested that social environments that thwart satisfaction of these needs cause less optimal forms of motivation and have harmful effects on well-being. In the current study, we provide evidence supporting the premise that the levels of flourishing or well-being were higher when the psychological needs were more satisfied in an exercise context. In general, the results of previous studies [3,37,38,39] showed that need satisfaction and well-being were positively and strongly associated. Milyavskaya and colleagues [38] noted that need satisfaction at different levels of experience can affect changes in well-being.

The current results confirm that levels of psychological need satisfaction specific to exercise are related to higher levels of PA, and lower levels of NA. Gunnell et al. [17] stated that psychological need satisfaction positively correlated with PA and negatively correlated with NA. Similarly, McDonough and Crocker [18] reported that three basic needs were associated with PA and NA. Moreover, in the results of a study by Edmunds, Ntoumanis, and Duda [40], the researchers reported that psychological need satisfaction negatively correlated with NA. Wilson et al.’s [11] study has supported the link between greater satisfaction of SDT-based needs with enhanced PA and reduced NA in exercise settings. Thus, studies have shown that there is a clear link between the satisfaction of the basic psychological needs and PA/NA. Thereby, the results of our study are consistent with previous studies about affect.

The results for RAI demonstrated that as the fulfillment of psychological needs increased, self-reported RAI also increased. Previous studies pointed out that basic need satisfaction positively relates to adaptive psychological and behavioral responses. For example, McDonough and Crocker’s [18] study showed that there is a positive correlation between three basic needs and RAI. Also, Weman-Josefsson, Lindwall, and Ivarsson [22] found that the path from need satisfaction to autonomous motivation was strongly positive and significant. Edmunds and colleagues [40] found that positive correlations existed between the psychological needs and self-determined forms of motivational regulation. Similarly, Wilson, Rodgers, Blanchard, and Gessell [41] stated that the needs for competence and autonomy positively correlated with more self-determined exercise regulations. Gourlan, Trouilloud, and Sarrazin [35] reported that satisfaction of autonomy and relatedness needs positively associated with autonomous forms of motivation. The results of their study also revealed that the more the needs for autonomy and relatedness were satisfied, the more the individuals tended to be motivated towards physical activity. In another study [12], the researchers reported that need satisfaction positively associated with autonomous motivation and well-being. Gunnel et al. [42] found that psychological need satisfaction mediated the relationship between changes in autonomous motivation and well-being. 

Although we reported strong evidence concerning the relations of flourishing, affect, and relative autonomy to basic psychological need in adult exercisers in the present study, limitations exist. From a theoretical perspective, one could argue that higher levels of flourishing, PA, and RAI lead one to rate all life domains more positively. Thus, this study should have initially clustered on levels of flourishing. Variables such as PA and NA are historically inherent in temperament models [43] and point to the potential that more positive/less negative temperaments relate to rating oneself more positively. Additionally, when considering limitations, all data were self-reported. Indeed, future research should use objective measures of physical activity behavior. Another limitation concerns the broad age range of the participants. The participants were not allocated into groups according to their ages in this study. The classification of psychological needs in relation to excise according to different age groups could add a different dimension to the study.

In conclusion, keeping these limitations in mind, the results of the present study supported the notion that the fulfillment of exercise-specific basic psychological needs is important even in participants from the same environment (fitness centers). Importantly, this study brought flourishing into the exercise psychology literature. Lastly, it appears that health clubs serve as effective environments for some individuals to maximize the fulfillment of their basic psychological needs. Further exploration of how and why some participants reported their three basic needs to have been met is an important topic for future research.

## Figures and Tables

**Table 1:** Mean, standard deviation, and inter-correlations values for study variables.

Variables	M ± SD	1	2	3	4	5	6	7
1. Autonomy	4.10 ± 0.58	1.00	-	-	-	-	-	-
2. Competence	4.11 ± 0.59	0.81 **	1.00	-	-	-	-	-
3. Relatedness	4.03 ± 0.66	0.67 **	0.65 **	1.00	-	-	-	-
4. Flourishing	47.54 ± 5.91	0.53 **	0.57 **	0.47 **	1.00	-	-	-
5. PANAS PA	36.58 ± 6.57	0.38 **	0.43 **	0.34 **	0.42 **	1.00	-	-
6. PANAS NA	17.40 ± 6.02	−0.16 **	−0.17 **	−0.18 **	−0.23 **	−0.15 **	1.00	-
7. RAI	11.55 ± 4.28	0.42 **	0.43 **	0.34 **	0.37 **	0.28 **	−0.21 **	1.00

** *p* < 0.01. M: Mean; SD: Standard deviation; PANAS: Positive and Negative Affect Schedule; PA: positive affect; NA: negative effect; RAI: Relative Autonomy Index.

**Table 2:** Descriptive characteristics of participant characteristics and study variables by clusters with univariate, post hoc, and effect size calculations.

Variables	Cluster								
	1 (*n* = 233)	2 (*n* = 361)	3 (*n* = 182)	Univariate and Post hoc Results			Hedges’ *g* for Cluster Comparisons		
	M (SD)	M (SD)	M (SD)	F_(2, 786)_	*η* ^2^	Post hoc	1–2	1–3	2–3
Autonomy	4.75 (0.29)	4.02 (0.29)	3.44 (0.44)	807.80	0.68	1 > 2 > 3	2.51	3.59	1.67
Competence	4.76 (0.28)	4.04 (0.29)	3.44 (0.47)	816.90	0.68	1 > 2 > 3	2.51	3.51	1.66
Relatedness	4.75 (0.32)	3.96 (0.36)	3.26 (0.46)	814.30	0.68	1 > 2 > 3	2.29	3.84	1.76
Age	27.58 (8.28)	28.59 (8.57)	27.32 (8.39)	1.76	0.01	None	−0.12	0.03	0.15
BMI	23.31 (3.38)	23.29 (3.18)	23.32 (3.25)	0.01	0.00	None	0.01	−0.00	−0.01
Minute/week	289.98 (154.41)	263.74 (150.75)	238.85 (148.07)	5.89	0.02	1 > 3	0.17	0.34	0.17
Sport/years	8.14 (6.63)	6.83 (6.41)	5.40 (5.61)	9.66	0.02	1 > 2 > 3	0.20	0.44	0.23
Flourishing	51.44 (4.49)	47.42 (4.84)	42.80 (5.90)	152.30	0.28	1 > 2 > 3	0.85	1.67	0.88
PANAS PA	39.85 (5.92)	36.33 (6.14)	32.88 (6.11)	67.81	0.15	1 > 2 > 3	0.58	1.16	0.56
PANAS NA	16.27 (5.36)	17.51 (5.82)	18.63 (6.93)	8.04	0.02	1< 2, 3	−0.22	−0.39	−0.18
RAI	13.79 (3.08)	11.42 (3.74)	8.93 (4.99)	79.64	0.17	1 > 2 > 3	0.68	1.20	0.59

BMI: body mass index.
